# Case Report: A novel *DLL4* variant in a neonate with Adams-Oliver syndrome

**DOI:** 10.3389/fped.2025.1532561

**Published:** 2025-03-03

**Authors:** Yanping Huang, Jin Wang, Lingkong Zeng, Shi Wang, Xuechen Zhang

**Affiliations:** Department of Neonatology, Wuhan Children’s Hospital (Wuhan Maternal and Child Healthcare Hospital), Tongji Medical College, Huazhong University of Science & Technology, Wuhan, Hubei, China

**Keywords:** Adams-Oliver syndrome, *DLL4*, case report, new born, genetic disease

## Abstract

Adams-Oliver syndrome is a rare congenital disorder with six subtypes that have been identified. Subtypes 1, 3, 5, and 6 have an autosomal dominant inheritance pattern, whereas subtypes 2 and 4 have an autosomal recessive inheritance pattern. The clinical phenotype of Adams–Oliver syndrome is heterogeneous and can be accompanied by abnormalities in other organs, especially the cardiovascular system, such as cutis marmorata telangiectatica congenita, pulmonary hypertension, vascular abnormalities in other organs, and congenital heart defects. Herein, we report a case of Adams–Oliver syndrome caused by a *de novo* variant in *DLL4*. The patient was a neonate with clinical manifestations of skin defects who was diagnosed with Adams–Oliver syndrome on the basis of genetic testing.

## 1 Introduction

Adams‒Oliver syndrome (AOS) is a disease characterized mainly by hypoplasia of the skin and terminal transected limb defects that may be accompanied by abnormalities of the cardiovascular and central nervous systems (1, 2). AOS is a type of congenital skin dysplasia (ACC), the mechanism of which is not completely clear, and genetic defects are one of its important causes. The currently known disease-associated genes include the *DOCK6*, *ARHGAP31*, *RBPJ*, *NOTCH1* and *DLL4* genes (3–5). Because the clinical manifestations of AOS vary widely, treatment needs to be individualized. Comprehensive management of AOS is particularly important.

This article reports a case of AOS that was diagnosed and treated by our team and summarizes the clinical and genetic characteristics of the disease through a review of the literature, aiming to improve the understanding of the disease.

## 2 Case description

**Figure 1:**
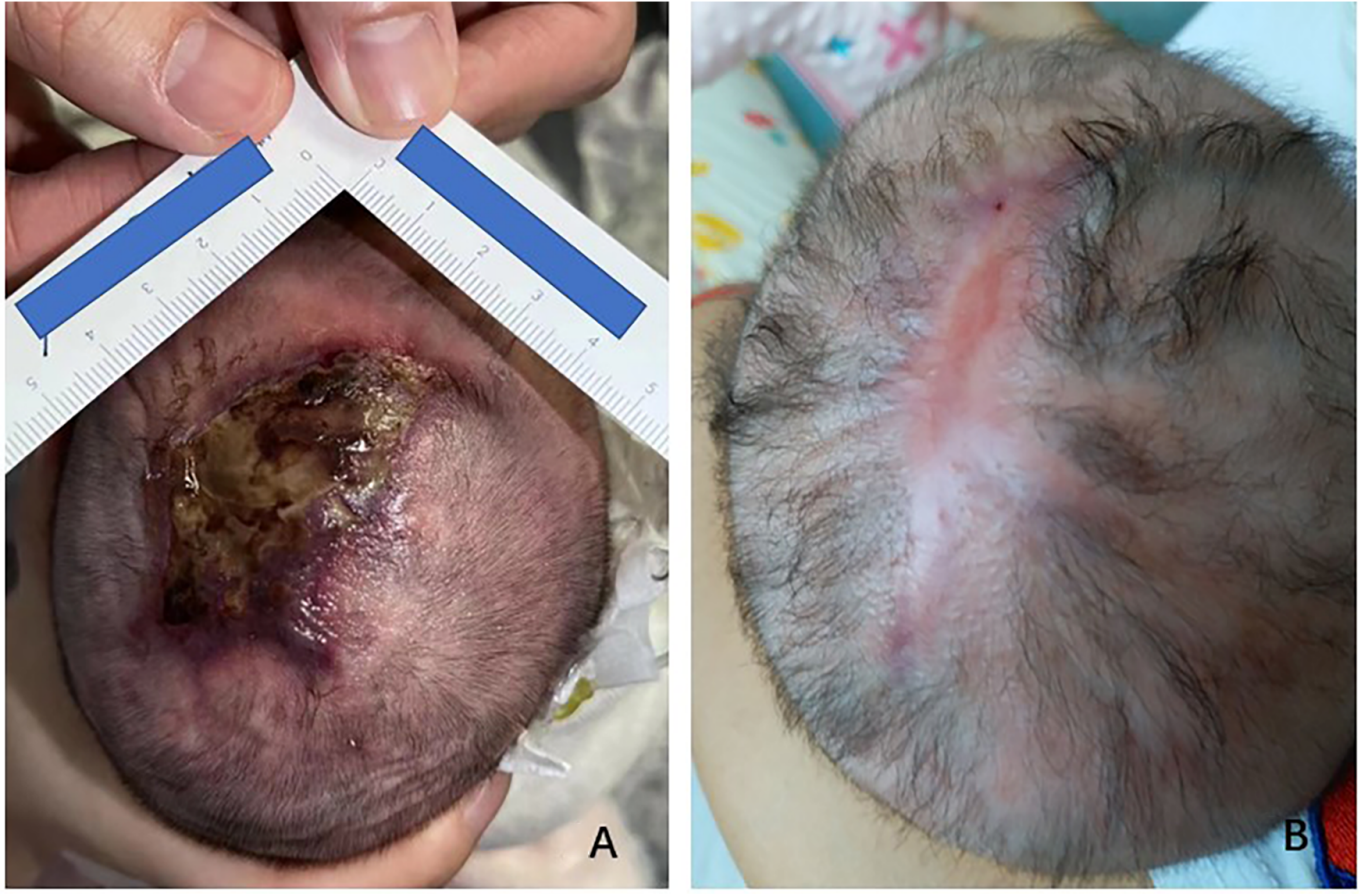
Skin manifestations on the top of the head before **(A)** and after treatment **(B)**.

The patient was a 7 h-old male born by cesarean section at 40 weeks' gestation, with a birth weight of 3,500 g. The Apgar scores at 1 min and 5 min were 8 and 9, respectively. The mother was healthy during pregnancy, with no abnormalities in the umbilical cord or placenta. After admission, an approximately 4.5*2.5 cm irregularly shaped area of skin loss was observed at the top of the head, and the surrounding skin was dark red (Figure 1A). All the fingers of both hands were shorter than other newborns of the same gestational age, and no other remarkable symptoms were observed.

Blood analysis revealed the following: white blood cell count, 21.84 × 10^9^/L; hemoglobin concentration, 198 g/L; hematocrit, 60.8%; platelet count, 267 × 10^9^/L; lymphocyte percentage, 20.8%; neutrophil percentage, 66.2%; monocyte percentage, 11.9%; and eosinophil percentage, 0.5%. The routine urine, stool, liver and kidney function, and electrolyte test results were normal. The C-reactive protein (CRP) level was 1.08 mg/L (0–3.0 mg/L), and the procalcitonin (PCT) level was 0.26 ng/ml. No pathogenic bacteria were detected in the blood or local secretion cultures. Tests for antibodies against cytomegalovirus, rubella virus, *Toxoplasma gondii*, herpes simplex virus, human immunodeficiency virus, hepatitis B virus, hepatitis C virus, and syphilis were negative. Head CT revealed a local defect at the top left of the scalp, slight thickening of the surrounding local soft tissue, no lesions with abnormal density in the parenchyma of the bilateral cerebral hemispheres, no expansion of the ventricular system, no ectopia in the midline structure, and no abnormalities in the skull bone window. Cardiac ultrasound revealed a patent ductus arteriosus (2.4 mm) and a central atrial septal defect (3.7 mm). A local skin biopsy of the scalp defect site revealed excessive keratosis of the squamous epithelium, thin epithelium, little inflammatory cell infiltration in the interstitium, necrosis, and calcified tissue. Ultrasound of the liver, spleen, and kidney revealed no abnormalities.

**Figure 2:**
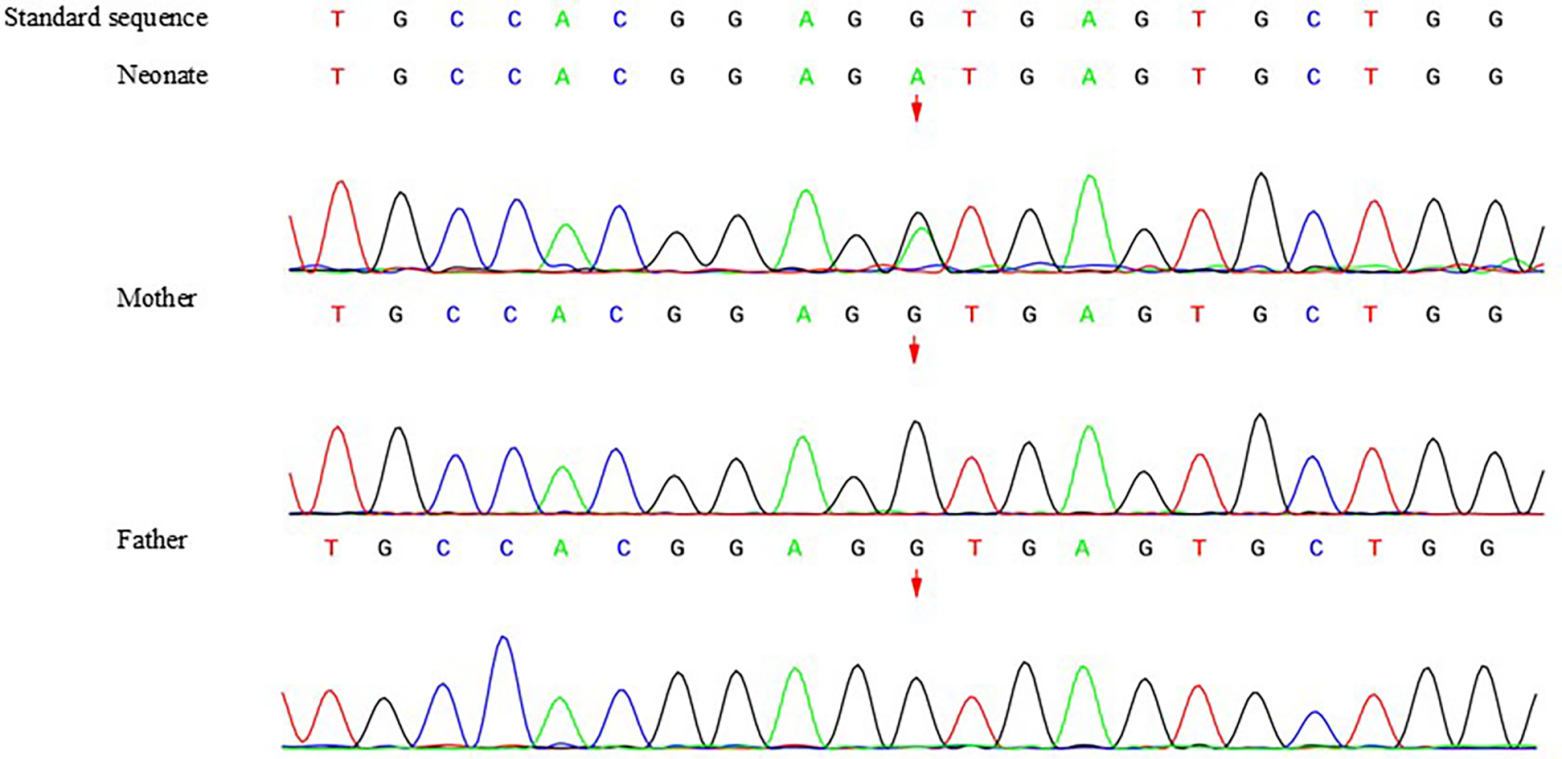
Sanger sequencing result of the whole family members. A heterozygous variant in *DLL4*(NM_019074.4): c.2052 + 1G > A, which is an intronic donor splice site variant, was detected in the infant, and neither parent carried the variant.

Trio whole-exome sequencing was performed for genetic testing in this study, and Sanger sequencing was performed for validation. A heterozygous variant in *DLL4*(NM_019074.4): c.2052 + 1G > A, which is an intronic donor splice site variant, was detected in the infant, and neither parent carried the variant (Figure 2). The variant was considered “likely pathogenic” according to the ACMG classification (6). The novel variant is a null variant (PVS1_Moderate); neither parent carried the mutation, which was considered to be a new mutation (PS2); and the incidence of variation in population databases is very low (PM2_Supporting). MaxEntScan was used for mRNA sequence prediction, this variant was predicted to affect the splice donor site and create a new splice donor. As a result, 86 bp may be inserted into the mRNA.

After admission, the child was treated with ampicillin and ceftazidime for anti-infection purposes (stopped after three days of continuous use), and topical drugs, including recombinant human epidermal growth factor topical solution (I) (Shenzhen Huashengyuan Bio, China), collagenase ointment (Liaoning Weibang Bio, China), and hydrocolloid dressing (Coloplast, Denmark), were applied after local debridement. Simultaneously, the scalp wound was contracted and folded for external fixation, and the wound gradually narrowed. After 2 weeks of hospitalization, the wound size was reduced to 2.5 cm*1.5 cm. After discharge, the external drugs were continued, and the wound had completely healed at 3 months after birth (Figure 1B).

## 3 Discussion and conclusion

ACC, also known as congenital absence of skin (CAS), refers to the absence of skin and/or subcutaneous tissue at birth; the cause of CAS is unclear and may be caused by intrauterine infection, chromosomal abnormalities, single-gene inheritance, or other reasons (1). Currently, nine types of congenital skin defects have been defined, including simple scalp congenital defects, scalp defects combined with limb abnormalities, congenital skin defects combined with papyriform fetuses, congenital skin defects combined with bullous epidermolysis, limb skin defects without bullous epidermolysis, congenital skin defects caused by intrauterine infection, congenital skin defects combined with other malformations, etc (1, 2). AOS is a type of ACC, and gene defects are important factors in AOS. The disease-associated genes identified to date include the *DOCK6*, *ARHGAP31*, *RBPJ*, *NOTCH1*, *EOGT* and *DLL4* genes (7). AOS can be divided into six types according to genotype: type 1, caused by *ARHGAP31* gene variation; type 2, caused by *DOCK6* gene variation; type 3, caused by *RBPJ* gene variation; type 4, caused by *EOGT* gene variation; type 5, caused by *NOTCH* gene variation; and AOS, caused by *DLL4* gene variation (7, 8).

The incidence of AOS is 1/225 000, and its main clinical feature is scalp dysplasia. Some children may have limb-end defects and cardiovascular development abnormalities. Defects of the autopod include brachydactyly, finger/toe oligodactyly, finger/toe syndactyly, finger/toenail dysplasia, and limb transection. Cardiovascular developmental abnormalities, including pulmonary hypertension, ventricular septal defects, tetralogy of Fallot, and abnormal development of the great arteries, have been detected (9). Terminal transverse limb defects are also more common in AOS caused by *DLL4* gene variation, with brachydactyly, finger/toenail dysplasia, and limb transection being the most common defects. The manifestation of limb abnormalities in this study was shortened fingers. In the literature, children with cardiovascular developmental abnormalities have been reported, one child with a ventricular septal defect and one child with a loss of the pulmonary artery branch (10, 11). After admission, the patient underwent a complete cardiac ultrasound examination, which revealed the presence of a patent ductus arteriosus and central atrial septal defect. Reexamination via cardiac ultrasound at 3 months after birth revealed that both the ductus arteriosus and central atrial septal defect were closed, and no other obvious developmental abnormalities of the cardiovascular system were observed. In addition, two children with AOS were found in the literature, both of which presented with marbling skin changes, suggesting that skin manifestations should also be considered in children with AOS caused by a *DLL4* gene variant (12, 13).

With increasing research, a preliminary understanding of AOS pathogenesis has been obtained. Among the genes associated with AOS, the *NOTCH1*, *EOGT* and *RBPJ* genes are members of the NOTCH signaling pathway, which is widely involved in the differentiation of neuronal stem cells, hematopoietic stem cells, and other important tissue differentiation processes (14–16). The NOTCH signaling pathway is composed mainly of NOTCH receptors, NOTCH ligands, DNA-binding proteins, and other effector and regulatory molecules. NOTCH receptors include NOTCH1–4, and NOTCH ligands are transmembrane proteins that include multiple regions, such as the N-terminal region, delta/serrate/lag-2 (DSL) region, epidermal growth factor (EGF)-like region, transmembrane region, and intracellular region. Activation of the NOTCH signaling pathway is affected mainly by serrate-like ligands (Jagged 1, Jagged 2) and delta-like ligands, and the DLL4 protein encoded by the *DLL4* gene is a member of the delta ligand family. Therefore, the *DLL4* gene plays an important role in the formation and differentiation of blood vessels (17–19).

The treatment of AOS focuses on the treatment of skin defects and other symptoms, including infection prevention and the use of topical drugs (3, 7, 11). The drugs used in other studies include recombinant human epidermal growth factor, convalescent ointments, and collagenase ointment (10–13). Owing to the difference in the area of skin lesions, recovery time varies greatly. In this study, recombinant human epidermal growth factor and collagenase ointment were used to treat skin defects, which were completely healed after continuous treatment for 3 months after birth. Other symptoms and signs should be treated according to the various clinical manifestations in children. Limb abnormalities and cardiac dysplasia are the most common abnormalities (10–13). Limb abnormalities can be treated with plastic surgery and functional exercise, whereas cardiac dysplasia often requires surgical treatment to improve cardiac function (1, 11). There are many types of abnormal cardiac development, including atrial septal defects, ventricular septal defects, and the absence of pulmonary arteries. In this study, no significant cardiac abnormalities were observed in the children.

The clinical manifestations of AOS are diverse, and the causative genes are different. Therefore, in clinical practice, genetic testing should be conducted in a timely manner for children with symptoms such as skin hypoplasia and terminal transection limb defects, and the test should focus on multiple genes, including the *DLL4* gene, for early identification. The treatment of AOS caused by the *DLL4* gene mainly involves infection prevention and topical drug use, and the therapeutic effect in most children is reasonable; for children with congenital heart malformations, early identification and surgical treatment should be performed to alleviate the relevant clinical symptoms (11–13).

In summary, AOS caused by the *DLL4* gene mainly manifests as skin hypoplasia, and some children may have terminal transection limb defects and congenital heart malformations. In children with similar clinical manifestations, genetic testing should be performed as soon as possible to identify the cause.

## Data Availability

The datasets presented in this study can be found in online repositories. The names of the repository/repositories and accession number(s) can be found in the article/Supplementary Material.
